# Hospital and Institutional News

**Published:** 1919-08-23

**Authors:** 


### HOSPITAL AND INSTITUTIONAL NEWS.

### BIRTHDAY HONOURS.

The following names appear among the Birthday Honours issued on Wednesday, August 13:—*Baronet*: Lieut-Col. Harry Gilbert Barling, C.B., M.B., F.R.C.S., Vice-Chancellor of Birmingham University, on whom this honour has been conferred for public services; Laurence Richard Philipps, J.P., founder of Paraplegic Hospital in Wales, on whom a similar honour has been conferred for public and local services. *Knight*: Dr. Robert Charles Brown, M.P., F.R.C.P., F.R.C.S., consulting medical officer of Preston Royal Infirmary, on whom this honour has been conferred for public and local services. Dr. Brown founded a scholarship for research at Cambridge, and has done much to promote infant welfare; John Young Walker MacAllister, F.S.A., F.R.G.S., President of the Library Association and Secretary of the Royal Society of Medicine for the last thirty-two years; this honour has been conferred for public services. Mr. MacAllister formed, and acted as secretary of, the War Office Surgical Advisory Committee, and organised the R.A.M.C. Bureau as well as Emergency Aid Corps for the Admiralty, War Office and Metropolitan Police; Henry Francis New, Mayor of St. Marylebone 1917-18, and Vice-President of the Marylebone War Supply Hospital; he has been chairman of many war committees; honour conferred for public and local services; William Ireland de Courcy Wheeler, M.D., F.R.C.S.I., member of the Consultative Committee of the War Office, and surgeon to the household of H.E. the Lord Lieutenant of Ireland. The honour of knighthood has been conferred for public services during the War.

### MALARIA IN ENGLAND.

Although a matter already several times mentioned of late in the Medical Press and giving rise as recently as last week to criticisms in the *Times*, which urges a still further educational campaign in trench and tropical fevers, it may, in sympathy with this last view, not be out of place to repeat briefly the arrangements already made by the Ministry of Health to assist practitioners in making their diagnosis. Blood specimens sent to them for diagnosis of malaria will be examined and reported upon free of charge. The methods of collecting are described on page 11 of "Suggestions for the Care of Malaria Patients," a copy of which has been supplied to all registered medical practitioners in England and Wales. The samples should be sent to Ministry of Health, Whitehall, S.W., marked outside "*Malaria Specimens, Urgent*," and accompanied by details and addresses. It is also to be remembered that in accordance with the Public Health Regulations, 1919, practitioners should notify cases of malaria to the medical officer of health for the district.

### POLAND AND THE MINISTRY OF HEALTH.

The Minister of Health of Poland has asked for the assistance of the International League of Red Cross Societies in connection with the suppression of typhus fever in that country, and in providing measures against the spread of typhus and other epidemics across Western Europe. The League of Red Cross Societies has appointed a Commission to investigate the conditions in Poland and to report to the League as to the measures that might advisedly be taken. Dr. Addison, Minister of Health, as the authority responsible in this country for national and international health questions, at the request of the League, has lent the services of Dr. G. S. Buchanan, C.B., one of the senior medical officers of the Ministry. Dr. Buchanan has accordingly been appointed a member of the Commission, which will leave shortly for Poland. The other members of the Commission are M. Castellani, an Italian bacteriologist; Colonel Cumming, of the Federal U.S.A. Medical Service; and M. Dopter, a French epideminologist.

### RESEARCH AT CAMBRIDGE.

Those who have made application have now been reappointed for the unexpired period of their Beit Memorial Fellowships for Medical Research, and the next election of Fellows will take place in December 1919. All these Fellowships were resigned shortly after the outbreak of war, and, the unexpended income having been meanwhile invested, it has been possible to increase the value of all the Fellowships from £250 to £300 per annum. Also regulations have been so modified that holders of teaching posts may become Fellows, provided that the director of the laboratory concerned is satisfied that their work permits of adequate time being devoted to research. Dr. A. E. Shipley, F.R.S., Master of Christ's College and Vice-Chancellor of the University of Cambridge, has been appointed a trustee, and Dr. C. H. Browning, director of the Bland-Sutton Institute of Pathology, has been appointed a member of the advisory board. All correspondence of candidates and Fellows should be addressed to the Hon. Secretary, Beit Memorial Fellowships for Medical Research, 35 Clarges Street, W. 1.

### EDUCATION GRANTS FOR EX-SERVICE MEN.

Under the Government scheme of financial assistance for the higher education of ex-service officers and men, the total number of grants awarded by the Board of Education now amounts to 5,400, including officers and men in about equal proportions. The courses in respect of which grants have been awarded include more than 1,000 for engineering and technological subjects, between 600 and 700 for classics, philosophy, and literature, and about an equal number for pure science and mathematics. Applications are still being received in large numbers, and are being dealt with at the rate of more than 200 a day.

### DENTAL TREATMENT.

In view of the facts that in expert dentistry we have an invaluable means of preventing ill-health, and that every possible means should be taken to enlighten the public as to the need of treatment for diseased teeth, the British Dental Association has formulated a scheme for the complete public dental service, which is now under consideration by the responsible authorities. It would probably be advisable to form a special dental section of the Ministry of Health under a dental director, but in any case we understand that the present scheme includes provision for dental treatment of (1) expectant mothers, and children under five years, (2) all children of school age, (3) all adults, whether entitled to National Insurance benefits or not, (4) patients suffering from tuberculosis and venereal disease—this as an essential to their cure. These are worthy and far-reaching objects, and must inevitably involve the most careful and extensive organisation. So invaluable to national health will success on these lines prove to be that we hope full consideration to detail will be given, and perfection not marred by incomplete and rushed legislation.

### TOO MANY DOCTORS?

Taking the instance of Glasgow University, we note that in this medical school the removal of the restricting influences of war has resulted in such a large enrolment of first-year students for the 1919 summer term, which began in April, that it has been found impossible to provide accommodation for additional students who may wish to commence in October next. No further applications for admission to first-year classes can be received until two months before the opening of the summer session on April 21, 1920, when the next five years' course of medical study will begin. We have heard similar reports of enormously increased student entry from several quarters, and it is probable that the direct effect of war experience has been to prompt many who would otherwise have taken up arts to give their attention to the medical profession. There is a warning to be taken from this situation before more young men turn to doctoring, and particularly to those of the last-mentioned group, for, although considerable developments under the Ministry of Health may be expected, it is questionable whether the medical scope will be twice what it was before the war. We must remember that since that time the number of medical students *has* practically doubled itself.

### RATS AS CARRIERS.

The City Corporation have published a report giving the results of investigations carried out upon the protozoal parasites of the rat, with special reference to the occurrence of spirochætal jaundice in man, and to the London rat as a natural reservoir of that disease. Thanks chiefly to the discoveries by Japanese workers, it has recently been demonstrated that the rat is a carrier of spirochætal jaundice and rat-bite fever, which though harmless to the rat, are pathogenic in man. Already, apart from Japan, infected rodents have been demonstrated in America, France, Belgian, and North Africa. During the recent English investigation of the rats in this country four were found to be carriers out of one hundred and one caught. The report is well illustrated and makes most interesting reading.

### SUICIDE AND IMITATION.

Two City juries recently added unanimous riders to their verdicts in three cases of suicide by those throwing themselves before trains at the Blackfriars Underground District Railway station, and, in a third case, by placing the head in a gas-stove with taps turned on. The coroner stated that both these methods were becoming increasingly common, due, he thought, to the magnetic influence in large measure of imitation by suggestion, particularly in the case of those whose minds had been by some means or other weakened or unbalanced. Such action was put in train by the reading of letters and other gruesome and sensational details in the newspapers, which were greedily devoured by a section of the public and Press. The coroner's practice was, as in the cases before them, to pick out to read, maybe, a material line or lines only, culled from the letters or documents found on the bodies of deceased. The letters themselves—in their entirety, were passed by him to the jurors and lawyers interested in the case for their perusal, but kept from the reporters present in court. The coroner, in general, was strongly in favour of publicity at the inquests, including the presence of juries, who formed an important section of the public. If, however, it were advisable to sit on any class of case with Press excluded, but with a jury, he thought it was so in a certain number of selected cases of suicide in which there was little, if any, doubt that life had been intentionally taken by the deceased person. In cases such as those in which any one's good name was at stake, however, it would be unwise to hold the inquest *in camera*. He failed to see what good came of going into irrelevant, scandalous and gruesome details not directly bearing upon the cause of death, which only led to harm both to the public and to the unfortunate relatives.

### THE HOUSING QUESTION.

We understand that already sites have been selected for 400,000 houses, but that the time of their building is still a matter of doubt. The Ministry of Health is apparently attempting directly and indirectly to bring about a general speeding-up and would seem to lay blame for delay upon the local authorities, but one can well realise that these local bodies are chary of entering upon such essentially expensive undertakings. In spite of the recent official cry for economy, it is to be noted that in the Ministry's magazine of *Housing* it is suggested that to manage these affairs there are required a production officer, who will work in liaison with officials from the Ministry of Munitions for materials, the Ministry of Labour and the Ministry of Ways and Communications for transport, and also a regional advisory committee. At present there appears to be no information as to the exact source of supply for the considerable amount of money which all these bodies must certainly demand, and it seems impossible to expect local authorities to accelerate the process of linking-up with this army of officials. The taxpayers, like ourselves, anxiously await further details as to the manner in which the official houses are to be built, for the medical interest in housing by no means ends with the customary decision of simple questions of site, aspect, and drainage systems.

### HOUSING PROBLEMS IN INNER LONDON.

Housing problems in the inner boroughs of London are rendered acute by the lack of land and because such land as is available is being rapidly acquired for warehouses and factories. Moreover, the price of land prohibits the erection of houses that can be let at a rent within the reach of a middle-class working family. Therefore, if a reasonable profit is to be made it can only be by building the enormous blocks of flats which have sprung up everywhere during the last decade. Dr. G. B. Millson, Medical Officer of Health for Southwark, in his 32nd annual report, deals with the housing problem in what is a typical Thames-side borough. Despite the fact that there has been a serious shrinkage in the population in the last ten years, Dr. Millson states that there is at the same time serious overcrowding, which cannot be remedied. Southwark, he says, is one of the most densely populated districts in the Metropolis. There is little vacant land upon which to build new houses.

### THE PROSPECT OF IMPROVEMENT.

Dr. Millson states that there is a large number of houses in the old part of Southwark, notably the Borough at the foot of London Bridge, quite unfit for human habitation, which should be pulled down. The portion of the district between the Elephant and Castle and the river has been for years past acquiring a commercial aspect. This change appeared to be desirable from a health and a municipal point of view. It was therefore inadvisable in this ancient part of the borough to rebuild many of the old rotten houses and narrow courts which, in a few years, must give place to large warehouses, factories, and offices. The housing problem in relation to those families who cannot pay an economic rent was a very difficult one to solve. Whatever form a subsidy should take, or by whom it should be paid, something of the kind was absolutely necessary if the housing conditions of the poorest of the people were to be improved. In a special report to the Council on the problem, Dr. Millson states that there are 202 houses now unoccupied which could be repaired and made habitable for occupation, which would find accommodation for over 1,600 people. The Council have decided to see that these are either repaired by the owners or by themselves. The Ministry of Health has undertaken to convert empty houses into flats wherever that can be done. On the Duchy of Cornwall estate there will soon, it is hoped, be a movement that will eventually accommodate some hundreds of people, and other building schemes in Southwark, delayed by the War, are about to begin.

### A CHANGE OF POLICY AT CARDIFF.

King Edward VII.'s Hospital, Cardiff, has begun to feel the loss of Colonel Bruce Vaughan, from which we draw the inference that the tributes to his work were not exaggerated. Now that he is dead the institution finds itself in need of a leader to bear the responsibility which he carried. Everyone had come to rely on Colonel Bruce Vaughan for raising the necessary money, and now that his support is lost the magnitude of the difficulties ahead is clear. To begin with, in spite of additions, the waiting list is 1,200. Much more money is needed, and now that the hospital has lost its leader it is being asked whether the whole task is not more than the institution can shoulder. As General Lee has pointed out, a new policy is demanded to decide whether the municipality of Cardiff should not be asked to provide some of the beds which the city needs, whether local hospitals could not be established so as to limit the wide district from which patients come, and to inquire if any way is possible to admit more in-patients than at present. It is also suggested that the investments should be revised to secure a larger income. These plans and proposals reveal the loss of the old hand upon the tiller; but King Edward VII.'s Hospital has accomplished so much that it can be excused for considering what is the limit of its capacity unless it is much better supported than at present.

### SIR WILLIAM OSLER'S UNCLE.

An interesting series of the letters of a London medical student commences in the August number of *Guy's Hospital Gazette*. The author of them is Dr. Edward Osler, the uncle of Sir William Osler, who "walked" Guy's Hospital in 1816. Dr. Edward Osler, after he served for seven years as house surgeon at the Swansea Infirmary, practised for many years in Truro. He was a very versatile man, and contributed papers to the Royal Society on the habits of marine animals, which he studied when making a special trip to the West Indies, commemorated in a poem called "The Voyage" published in 1830. The first extract from the letters is perhaps too grisly for general reading, and deals with the study of operative surgery at Guy's Hospital one hundred years ago.

### HOSPITAL RESIDENT MEDICAL OFFICERS: SUPPLY AND DEMAND.

The advertisement columns of the medical journals are always a good indication of the condition of the "labour market" for hospital officers. Just now salaries are being offered to house surgeons and physicians such as have never before been heard or dreamed of, from which an acute shortage of resident medical officers can be inferred, as indeed is known to be the case. One of the most curious examples of this shortage, and of the straits to which hospitals are in consequence reduced, is an advertisement which recently offered a salary of £200 for a resident male house surgeon, with the alternative offer of £180 a year for an (unqualified) fifth year student! To say that the authorities of this hospital assess the difference between a qualified and an unqualified man at only £20 a year would scarcely be fair, though *primâ facie* it might seem arguable. The really astonishing thing is that this salary (with board-residence, of course) should be obtainable by a student not yet qualified, as such it is indubitably a sign of the times.

### A JUDGE'S STRICTURE ON DOCTORS.

In dealing with the medical evidence at a workman's compensation case, Judge Rowland Rowlands made the following severe criticism of the medical evidence: "Not one of the doctors," he said, "made a single note of what was done at the time, and here am I, trying the case, who have to conclude that one or the other is telling a lie. Here, too, is a hospital, where things are supposed to be kept, of the very thing that is now said to be the cause of death, there is not a single mention." In defence of the Swansea Hospital, Mr. Metcalf said that the hospital was very short-staffed. The Judge replied that similar excuses were made for the hospitals in the colliery districts until he insisted that notes of accident cases should be kept. The matter was important, because "all these accident cases come into court at one time or another." We know that casualty departments are often very busy, but the making of notes should be the rule. The real difficulty is to get the doctors to acquire the habit. Once the habit is acquired, time is always found for making records. Where they are not made, and medical evidence is later called for, the doctors cannot defend themselves against the charge of falsehood, which was made on the present occasion.

### A CONTRAST IN ECONOMY.

We are glad to see that Walsall Hospital, which, on the whole, has been in luck's way during the past few years, has now received an important gift from the local branch of the Red Cross Society. This has presented to the General Hospital the entire stores, supplies, surgical appliances and dressings which remained over when the Society's own hospital was closed. The value of the gift is estimated to be over £1,000. But more hangs on this and similar gifts than the generosity of the donors. If the Government's stores had been as economically disposed of as those of the Red Cross Society, what waste would have been avoided! The Red Cross, we make no doubt, could have sold its surplus to advantage, and the Government, which does not give, cannot even sell them without confusion. There is an evil attending State enterprise from which nothing can save it, whereas voluntary agencies can escape. If the winding-up of the Red Cross stores department is compared with the Government's attempts in the same department, the contrast is manifest.

### ENFIELD HOSPITAL AND THE CHARITY COMMISSIONERS.

There is no settlement of the dispute between the medical staff of Enfield Cottage Hospital and the governors, notwithstanding the fact that a special meeting of the latter was called to decide the matter. No sooner were the doctors outvoted on one of their resolutions than they left the meeting, which proves our original contention that they were endeavouring to override any authority but their own. The dispute turns on the question whether the power to admit or reject cases shall rest, as in the rules, with the council, or, as the medical staff has desired, with the doctors. With regard to the future, Mr. F. W. Fletcher, who for ten years was chairman of the late council, remarked that the council would decline office if some of the present medical staff remained. The trustees cannot continue without a council. They will have to appeal to the Charity Commissioners, and it has been decided to send a report to the Commissioners. It must be clear to any impartial observer that the doctors have given themselves away by refusing to accept the decision of the governors to whom they referred their case.

### MARRIED WOMEN DOCTORS.

Portsmouth has been concerned about the idea of a married woman doctor being appointed to the Municipal Maternity Hospital. The Maternity and Child Welfare Committee proposed the appointment of Dr. Mabel Ross, but was asked to reconsider the matter. The Committee has done so, but now report strongly in favour of the appointment. In order to meet the objection raised by the Council to a married woman holding the appointment, the Committee suggests that in the first place the appointment might be made for twelve months only, to be reconsidered at the end of that period if it is found that for any reason domestic claims upon Dr. Ross prevent her carrying out efficiently the duties of her appointment. The Committee points out that Dr. Ross has already for over a year carried out satisfactorily the duties of medical officer to the Child Welfare Centres, and expresses the opinion that for a maternity hospital the most suitable medical officer is a married woman.

### THIS WEEK'S DRUG MARKET.

The market continues quiet. It is as yet too soon to see the effect of the Premier's statement regarding the Government's interim trade policy, but it is to be noted that, although the restriction on imports is to come to an end on September 1, the importation of products associated with "key" industries may still be restricted. This restriction may affect a number of chemical products, so that the drug trade will not be altogether freed. Menthol and Japanese peppermint oil are still very dull and prices are barely maintained. There is a little more life in Japanese refined camphor. American peppermint oil is scarcer and dearer. Olive oil continues scarce and commands high prices. Santonin is again much dearer. Sugar of milk, thymol, saffron, lemon oil, clove oil, orange oil, and bergamot oil have an upward price tendency. Carbolic acid is dearer, and the values of salicylates are firmly maintained. Supplies of phenacetin are inadequate and the price is higher. On the other hand, antipyrin is more plentiful and lower.

